# Restoring cerebellar-dependent learning

**DOI:** 10.7554/eLife.100251

**Published:** 2024-07-16

**Authors:** Jessica L Verpeut

**Affiliations:** 1 Department of Psychology, Arizona State University Tempe United States

**Keywords:** neural plasticity, Fragile X Syndrome, LTD, cerebellum, Purkinje cells, LTP, Mouse

## Abstract

Behavioral and pharmaceutical interventions reverse defects associated with increased cerebellar long-term depression in a mouse model of Fragile X syndrome.

**Related research article** Shakhawat AM, Foltz JG, Nance AB, Bhateja J, Raymond JL (2023) Systemic pharmacological suppression of neural activity reverses learning impairment in a mouse model of Fragile X syndrome. *eLife* **12**:RP92543. doi: 10.7554/eLife.92543.

Neurodevelopmental disorders, such as Fragile X syndrome, are often characterized by neurons connecting incorrectly, leading to impairments in learning, memory and cognitive flexibility (Schmitt et al., 2022). Yet the brain can reorganize itself in response to an organism’s changing needs by altering the strength and number of connections (or synapses) between certain neurons. Whether therapeutics could restore behavioral deficits by encouraging nerve cells to 'rewire,' particularly when the brain is at its most malleable, remains a controversial question. In fact, enhancing neural plasticity has been shown to sometimes impair learning (Navakkode et al., 2022; Makin and Krakauer, 2023; Diniz and Crestani, 2023).

Long-term depression (LTD for short) is a type of plasticity that results in weaker synapses. Perhaps counterintuitively, this process is crucial to fine-tune neural responses and enable learning, memory and cognitive flexibility. For example, this is the case in the cerebellum, the brain center best known for controlling movement and learning new motor skills. There, output neurons, known as Purkinje cells, process and integrate the information received from parallel and climbing fibers originating from cells in other parts of the cerebellum or the nervous system. Both types of fibers work together to regulate the strength of the synapses between parallel fibers (PF for short) and Purkinje cells via LTD (Ito, 1989).

Oculomotor learning is a cerebellar mechanism by which the brain can use previous experiences to fine-tune how eye muscles compensate for head movements so that vision can remain steady (a process known as the vestibulo-ocular reflex, or VOR; Nguyen-Vu et al., 2017). Mice with increased LTD at the PF-Purkinje cell synapses have disrupted oculomotor learning, but why this is the case has remained unclear. Now, in eLife, Amin Shakhawat, Jennifer Raymond and colleagues at Stanford University report how this defect can be reverted in a mouse model of Fragile X syndrome (Shakhawat et al., 2023).

Scientists often use the VOR to examine cerebellar function and synaptic plasticity, as animals can be trained to adjust the strength of this reflex (Hirano and Inoshita, 2021; Ito, 1989; Clopath et al., 2014). For instance, mice trying to keep track of rotating black and white stripes while moving on a turntable will make smaller eye movements if this stimulus and the head are moving in the same direction (VOR-decrease learning). Conversely, these eye movements will increase if the stripes and the head travel in opposite directions (VOR-increase learning).

VOR-increase training relies heavily on LTD, yet studies in mice show that such learning is impaired when this type of plasticity is enhanced at PF-Purkinje cells; on the other hand, VOR-decrease learning, which is less dependent on long-term depression, is preserved. This led Shakhawat et al. to propose that enhanced LTD can be too easily triggered by erroneous signals; the cerebellar circuit responds by raising the threshold required to activate this plasticity, which then makes it more difficult for LTD to be recruited during learning.

To test this hypothesis, the team used mice in which Purkinje cells carry the mutation responsible for Fragile X syndrome – a genetic change known to enhance PF-Purkinje cell LTD. First, Shakhawat et al. confirmed that these animals (known as L7-*Fmr1* KO mice) showed profound impairment in their VOR-increase learning, while their VOR-decrease learning remained intact. Next, they tested whether two types of interventions that could prevent or reduce a prior increase in activation threshold for LTD might be able to reverse these defects.

The behavioral intervention consisted of both wild-type and L7-*Fmr1* KO mice receiving a VOR-decrease ‘pre-training’ session (which can improve LTD at PF-Purkinje cell synapses) before their VOR-increase training. This manipulation did not affect the control group, but it greatly improved VOR-increase learning in the mutated mice.

In the pharmaceutical intervention, Shakhawat et al. treated wild type and L7-*Fmr1* KO animals with diazepam prior to VOR as this compound is known to decrease neural firing in the cerebellum (and would therefore prevent incorrect LTD activation prior to testing). Similar to pre-training, diazepam temporarily rescued VOR-increase learning impairments in L7-*Fmr1* KO mice if administered 18-24hours before the task. The team further showed that diazepam could restore another type of LTD-dependent learning (known as the optokinetic reflex) for which these mice are also impaired. In fact, receiving this treatment led to the animals outperforming their wild-type counterparts.

Taken together, these results suggest that defects in VOR-increase learning can be rescued through specific pre-training or the use of certain pharmaceuticals (Figure 1). The findings support a model in which prior experiences reshape the activation thresholds of mice with enhanced LTD at their PF-Purkinje cells in such a way that subsequent activation of this plasticity is hindered.

**Figure 1.**
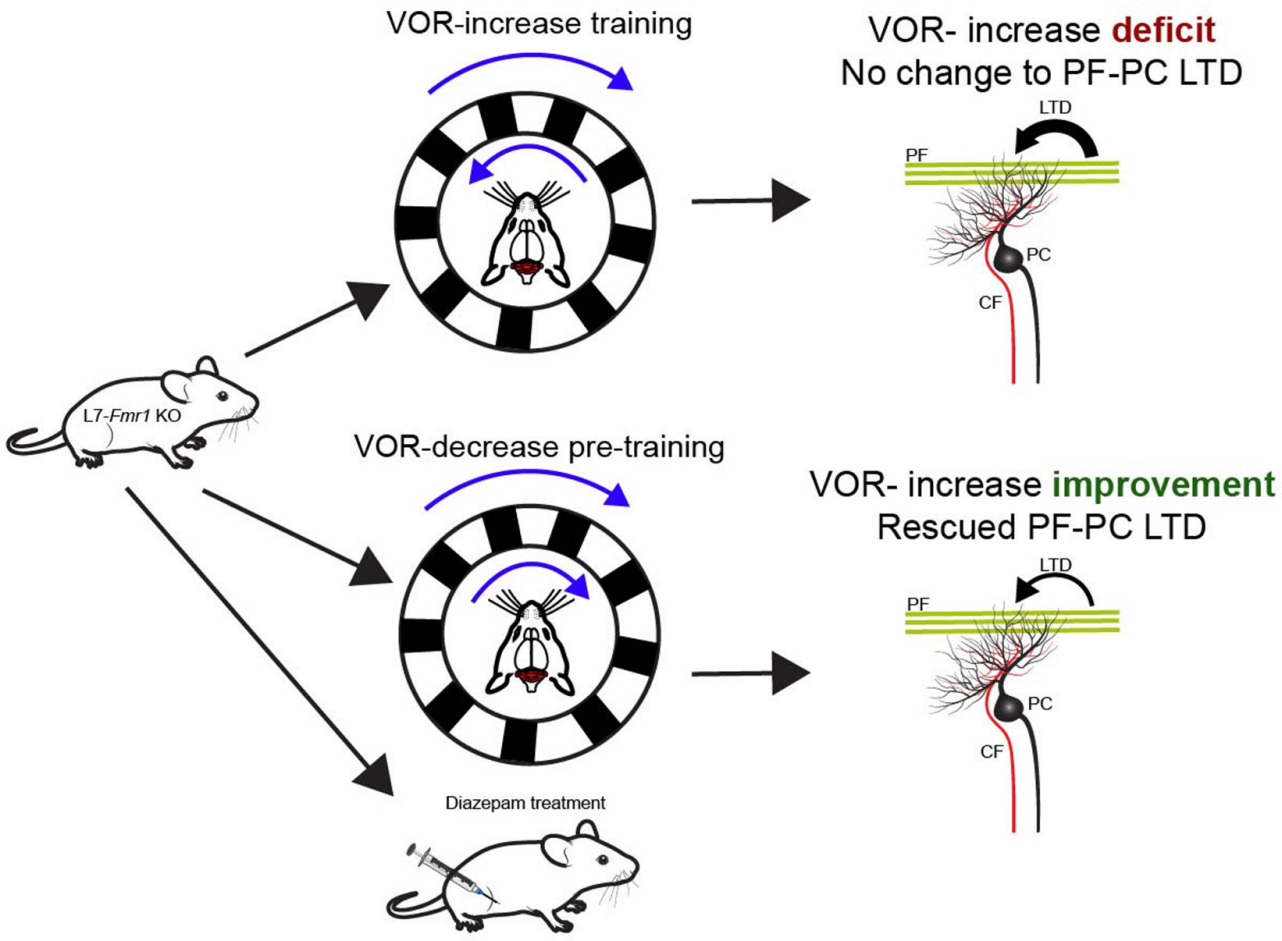
Behavioral and pharmaceutical interventions can rescue vestibulo-ocular reflex increase learning in a mouse model of Fragile X syndrome. Top: Genetically modified animals (L7-*Fmr1* KO mice) whose Purkinje cells carry the mutation that causes Fragile X syndrome show impairments when trained to enhance their vestibulo-ocular reflex (VOR-increase learning). This type of learning relies on the cerebellum (red brain structure). VOR-increase learning involves the animals keeping track of a stimulus (black and white stripes, top blue arrow) rotating in the opposite direction as head movement (bottom blue arrow). The defect is linked to the mice displaying increased long-term depression (LTD; thick black arrow) at the synapses between Purkinje cells (PC; black) and parallel fibers (PF; green). This type of neural plasticity is under the control of both parallel and climbing fibers (CF; red). Bottom: When the VOR-increase learning is preceded by VOR-decrease pre-training (whereby the visual stimulus and head rotate in the same direction) or administration of diazepam, PF-Purkinje cell long-term depression is normalized (thin black arrow) and VOR-increase learning is improved for stimuli rotating at high frequencies.

Image credit: Mouse by Ethan Tyler and Lex Kravitz (CC BY 4.0), mouse head by Luigi Petrucco (CC BY 4.0), Purkinje cells and climbing fibers by Akshay Markanday (CC BY 4.0).

Alternative biological mechanisms may also be involved, however. For instance, the interventions rescued VOR-increase learning when the stimuli used during training were rotating at high frequencies (1Hz), but not at low frequencies (0.5Hz) – possibly because the processing of such frequencies does not rely as much on long-term depression. The mutation associated with Fragile X syndrome may lead to cellular changes or disruptions that were not assessed in this study, and further work should examine whether the structure of Purkinje cells in this mouse model remains unchanged. Follow-up research should also review how these local changes may reshape the long-distance connections between Purkinje cells and neurons outside of the cerebellar microcircuit.

Overall, this work has exciting therapeutic implications for Fragile X syndrome while also expanding our understanding of cerebellar long-term depression. Such knowledge may be crucial as the cerebellum has been implicated in several neurodevelopmental and aging disorders, yet specific treatments that target this structure are still lacking (Wang et al., 2014; Arleo et al., 2024; Iskusnykh et al., 2024).

## References

[bib1] Arleo A, Bareš M, Bernard JA, Bogoian HR, Bruchhage MMK, Bryant P, Carlson ES, Chan CCH, Chen LK, Chung CP, Dotson VM, Filip P, Guell X, Habas C, Jacobs HIL, Kakei S, Lee TMC, Leggio M, Misiura M, Mitoma H, Olivito G, Ramanoël S, Rezaee Z, Samstag CL, Schmahmann JD, Sekiyama K, Wong CHY, Yamashita M, Manto M (2024). Consensus paper: Cerebellum and ageing. Cerebellum.

[bib2] Clopath C, Badura A, De Zeeuw CI, Brunel N (2014). A cerebellar learning model of vestibulo-ocular reflex adaptation in wild-type and mutant mice. The Journal of Neuroscience.

[bib3] Diniz CRAF, Crestani AP (2023). The times they are a-changin’: a proposal on how brain flexibility goes beyond the obvious to include the concepts of “upward” and “downward” to neuroplasticity. Molecular Psychiatry.

[bib4] Hirano T, Inoshita T, Mizusawa H, Kakei S (2021). Cerebellum as a CNS Hub.

[bib5] Iskusnykh IY, Zakharova AA, Kryl’skii ED, Popova TN (2024). Aging, neurodegenerative disorders, and cerebellum. International Journal of Molecular Sciences.

[bib6] Ito M (1989). Long-term depression. Annual Review of Neuroscience.

[bib7] Makin TR, Krakauer JW (2023). Against cortical reorganisation. eLife.

[bib8] Navakkode S, Zhai J, Wong YP, Li G, Soong TW (2022). Enhanced long-term potentiation and impaired learning in mice lacking alternative exon 33 of Ca_V_1.2 calcium channel. Translational Psychiatry.

[bib9] Nguyen-Vu TB, Zhao GQ, Lahiri S, Kimpo RR, Lee H, Ganguli S, Shatz CJ, Raymond JL (2017). A saturation hypothesis to explain both enhanced and impaired learning with enhanced plasticity. eLife.

[bib10] Schmitt LM, Arzuaga AL, Dapore A, Duncan J, Patel M, Larson JR, Erickson CA, Sweeney JA, Ragozzino ME (2022). Parallel learning and cognitive flexibility impairments between *Fmr1* knockout mice and individuals with Fragile X syndrome. Frontiers in Behavioral Neuroscience.

[bib11] Shakhawat AM, Foltz JG, Nance AB, Bhateja J, Raymond JL (2023). Systemic pharmacological suppression of neural activity reverses learning impairment in a mouse model of Fragile X syndrome. eLife.

[bib12] Wang SSH, Kloth AD, Badura A (2014). The cerebellum, sensitive periods, and autism. Neuron.

